# ^7^Be-recoil radiolabelling of industrially manufactured silica nanoparticles

**DOI:** 10.1007/s11051-014-2574-0

**Published:** 2014-08-02

**Authors:** Uwe Holzwarth, Elena Bellido, Matteo Dalmiglio, Jan Kozempel, Giulio Cotogno, Neil Gibson

**Affiliations:** 1 Nanobiosciences Unit, Joint Research Centre, Institute for Health and Consumer Protection, European Commission, T.P.500, Via Enrico Fermi 2749, 21027 Ispra, VA Italy; 2 Department of Radiochemistry, Faculty of Nuclear Sciences and Physical Engineering, Czech Technical University in Prague, Břehová 7, 11519 Prague 1, Czech Republic

**Keywords:** Nanoparticles, Nanomaterials, Radiolabelling, Proton irradiation, ^7^Be, Recoil, Lithium compounds, Cyclotron

## Abstract

Radiolabelling of industrially manufactured nanoparticles is useful for nanoparticle dosimetry in biodistribution or cellular uptake studies for hazard and risk assessment. Ideally for such purposes, any chemical processing post production should be avoided as it may change the physico-chemical characteristics of the industrially manufactured species. In many cases, proton irradiation of nanoparticles allows radiolabelling by transmutation of a tiny fraction of their constituent atoms into radionuclides. However, not all types of nanoparticles offer nuclear reactions leading to radionuclides with adequate radiotracer properties. We describe here a process whereby in such cases nanoparticles can be labelled with ^7^Be, which exhibits a physical half-life of 53.29 days and emits γ-rays of 478 keV energy, and is suitable for most radiotracer studies. ^7^Be is produced via the proton-induced nuclear reaction ^7^Li(p,n)^7^Be in a fine-grained lithium compound with which the nanoparticles are mixed. The high recoil energy of ^7^Be atoms gives them a range that allows the ^7^Be-recoils to be transferred from the lithium compound into the nanoparticles by recoil implantation. The nanoparticles can be recovered from the mixture by dissolving the lithium compound and subsequent filtration or centrifugation. The method has been applied to radiolabel industrially manufactured SiO_2_ nanoparticles. The process can be controlled in such a way that no alterations of the ^7^Be-labelled nanoparticles are detectable by dynamic light scattering, X-ray diffraction and electron microscopy. Moreover, cyclotrons with maximum proton energies of 17–18 MeV that are available in most medical research centres could be used for this purpose.

## Introduction

The application range of manufactured nanomaterials is steadily increasing, and in parallel, there is growing concern about the safety of such materials. Since the interaction of nanomaterials with biological systems depends on a large number of parameters (Mailänder and Landfester 2009; Verma and Stellacci 2010), of which most are affected by the synthesis procedure (Vollath 2008; Marchisio et al. 2006), the hazard and risk assessment of industrially manufactured nanomaterials should at least include studies on materials as they result from their large-scale industrial manufacturing process. On the other hand, toxicity studies with such materials require reliable methods for detecting, tracing and quantifying nanoparticles (NPs), which is usually a major problem for non-labelled nanomaterials (Weiss and Diabate 2011). Radiolabelling represents one of the most sensitive and quantitative techniques for this purpose with the potential to properly quantify NPs in various biological systems (Pérez-Campaña et al. 2013; Llop et al. 2013; Pérez-Campaña et al. 2012; Weiss and Diabate 2011; Abbas et al. 2010; Ponti et al. 2009; Oughton et al. 2008; Kreyling et al. 2004). However, since radiotracers can usually not be incorporated into large-scale industrial manufacturing processes, post-synthesis radiolabelling procedures must be applied. Such procedures must minimise the risk of changing any of the NP properties that are relevant for their interaction with biological systems. Especially, surface modifications, e.g. using chelator molecules for attaching radiolabels, should be avoided especially for small NPs.

The possibilities and challenges of radiolabelling industrially manufactured NPs by post-synthesis irradiation with light ions or neutrons have been reviewed by Gibson et al. (2011), and engineering and procedural solutions have been reported to control temperature and to avoid thermally induced alterations of NP properties during proton irradiation (Holzwarth et al. 2012). An important finding when dealing with nuclear reactions in NPs is that the recoil energy of the created radionuclide is sufficient to eject the radiolabel from the NP in which it has been produced, and it may traverse many others before it is stopped in a more distant NP (Abbas et al. 2010; Gibson et al. 2011). Hence, radiolabelling of NPs by proton irradiation is recoil labelling, except for large NPs made of dense materials.^1^ For this reason, irradiation of NP suspensions will be inefficient because a large fraction of the radiolabels will be stopped in the liquid medium instead of being implanted in NPs. Recoil labelling can be used to radiolabel NPs such as SiO_2_, Al_2_O_3_ or carbon-based nanomaterials that do not offer adequate nuclear reactions producing radionuclides with properties suitable for radiotracing purposes under convenient irradiation conditions. This problem can be overcome by mixing the NPs to be labelled with a fine-grained compound acting as source of suitable radiolabels.

Soluble lithium compounds are an ideal source of radiolabels since ^7^Be, produced via the nuclear reaction ^7^Li(p,n)^7^Be, has a physical half-life *T*_1/2_ = 53.29 days (Firestone and Shirley 1999), which matches with the duration of most biodistribution or cell-uptake experiments (Gibson et al. 2011). The radiation emitted by the radiolabel should be easily detectable and quantifiable, ideally also suitable for imaging procedures. ^7^Be emits γ-rays with an energy of 478 keV and an abundance of 10.4 % (Firestone and Shirley 1999; National Nuclear Data Center 2014) and fulfils the basic requirements. Moreover, Be atoms are sufficiently small to be hosted as interstitial or substitutional impurities in the crystalline or amorphous structure of NPs of interest for risk assessment. The use of a soluble lithium compound offers the advantage of separating the NPs from residues of the lithium compound after proton irradiation by a simple washing procedure, followed by filtration and/or ultra-centrifugation.

In the present paper, a method able to provide ^7^Be-labelled NPs with activity concentrations that are sufficient for in vitro and in vivo studies without introducing significant changes of the NP properties as determined by dynamic light scattering (DLS), X-ray diffraction (XRD) and transmission electron microscopy (TEM) is presented. A simple model will be presented to estimate the activity concentrations to be expected in the labelled NPs which provide guidance to optimise the mixing ratio of lithium compound and NPs and the duration of the irradiations to achieve the desired batch sizes and activity concentrations of the NPs.

## Materials and experimental methods

### Production of ^7^Be and selection of the lithium compound

**Fig. 1:**
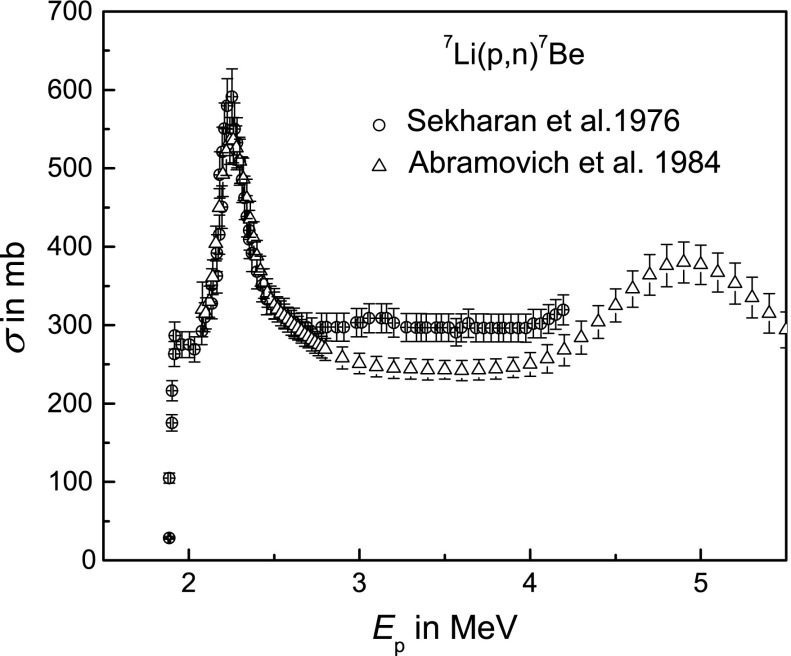
The reaction cross section *σ* of the nuclear reaction ^7^Li(p,n)^7^Be exhibits values of at least 250 mb throughout the proton energy range relevant for the present purpose and a narrow maximum of nearly 600 mb at around 2.25 MeV (data: Sekharan et al. 1976; Abramovich et al. 1984)

^7^Be can be produced via the reaction ^7^Li(p,n)^7^Be. Figure 1 shows the reaction cross sections^2^ (Sekharan et al. 1976; Abramovich et al. 1984; Gibbons and Macklin 1959) from the threshold energy *E*_thr_ = 1.88 MeV (National Nuclear Data Center 2014) up to 5.5 MeV. Around *E*_p_ ≈ 2.25 MeV, the excitation function exhibits a narrow maximum of the reaction cross section of nearly 600 ~ mb (Gibbons and Macklin 1959; Newson et al. 1957). Thus, ^7^Be can efficiently be produced already just above the reaction threshold.

The lithium compound should have a high number of Li atoms per unit mass in order to maximise the number of target atoms in the mixture of compound and NPs. In order to separate and recover the NPs from the mixture, the lithium compound should be soluble in water or other solvents such as ethanol. In this way, the NPs can be washed and recovered by filtration or centrifugation.

For NPs that may be attacked by strong bases, lithium compounds must be preferred which behave pH neutral in aqueous solution. Table 1 compiles some of the candidate compounds with their key properties. In spite of its unfavourable behaviour in water, LiH has been tested because it offers the highest number of lithium atoms per mass unit. LiH requires absolute ethanol as solvent because the reaction to lithium ethanolate, which is then soluble in ethanol, is much less violent than its reaction with water.

**Table 1:** Lithium compounds potentially useful for ^7^Be-recoil labelling experiments, with some of their key properties (data from Haynes and Lide 2010)

Lithium compound	Molar mass in g/mol	Bulk density in g/cm^3^	Li atoms per mg compound	Melting point in °C	Solubility, behaviour in water
LiH	7.95	0.78	7.57 × 10^19^	692	Violent reaction
Li_2_O	29.88	2.01	4.03 × 10^19^	1,430	Reacts with water
Li_3_N	34.83	1.27	5.19 × 10^19^	813	Violent reaction
LiCl	42.39	2.07	1.37 × 10^19^	610	Good, 845 ~ g/l

### Preparation of the nanoparticle lithium compound mixture

The lithium compounds were purchased from Sigma Aldrich. The SiO_2_ NPs were obtained from the JRC Nanoparticle Repository of representative industrial NPs. Among the very well-characterised NPs, the material *NM200* was selected, which is a precipitated synthetic amorphous silica that exhibits among the repository silica materials the smallest mean particle size. The polydisperse material consists of primary particles with a mean size of about 20 nm, which form larger aggregates with complex morphology and low to medium sphericity (Rasmussen et al. 2013). Analysis by TEM revealed that 88.7 % of the primary particles are smaller than 100 nm, 69.8 % smaller than 50 nm and 1.7 % are smaller than 10 nm (Rasmussen et al. 2013). BET yields a specific surface area of about 190 m^2^/g.

Nanoparticles and lithium compound have to be mixed in a predefined mass ratio. Mixing was carried out by hand in a glove box in a mortar using a pestle. The use of LiH requires a protective atmosphere since the substance is hygroscopic and tends to decompose into a LiH–LiOH–Li_2_O mixture. Therefore, LiH, Li_2_O or Li_3_N were mixed with NPs in a protective argon atmosphere. Mixing by hand requires at least 30 min before achieving an optically homogeneous mixture. Since NPs such as silica, α-and γ-alumina or nanodiamonds are harder than the Li compounds, the mixing procedure will also reduce the grain size of the lithium compounds by grinding.

Some modelling is required (see Sect. ‘Expected radiolabelling yield’) to optimise the mixing ratio between lithium compound and NPs since the flux of ^7^Be-recoil atoms that are available for labelling is increasing with the mass fraction of the lithium compound in the capsule, but at the expense of the number of NPs that can trap the recoils.

### Target system and irradiation capsule for proton irradiation

**Fig. 2:**
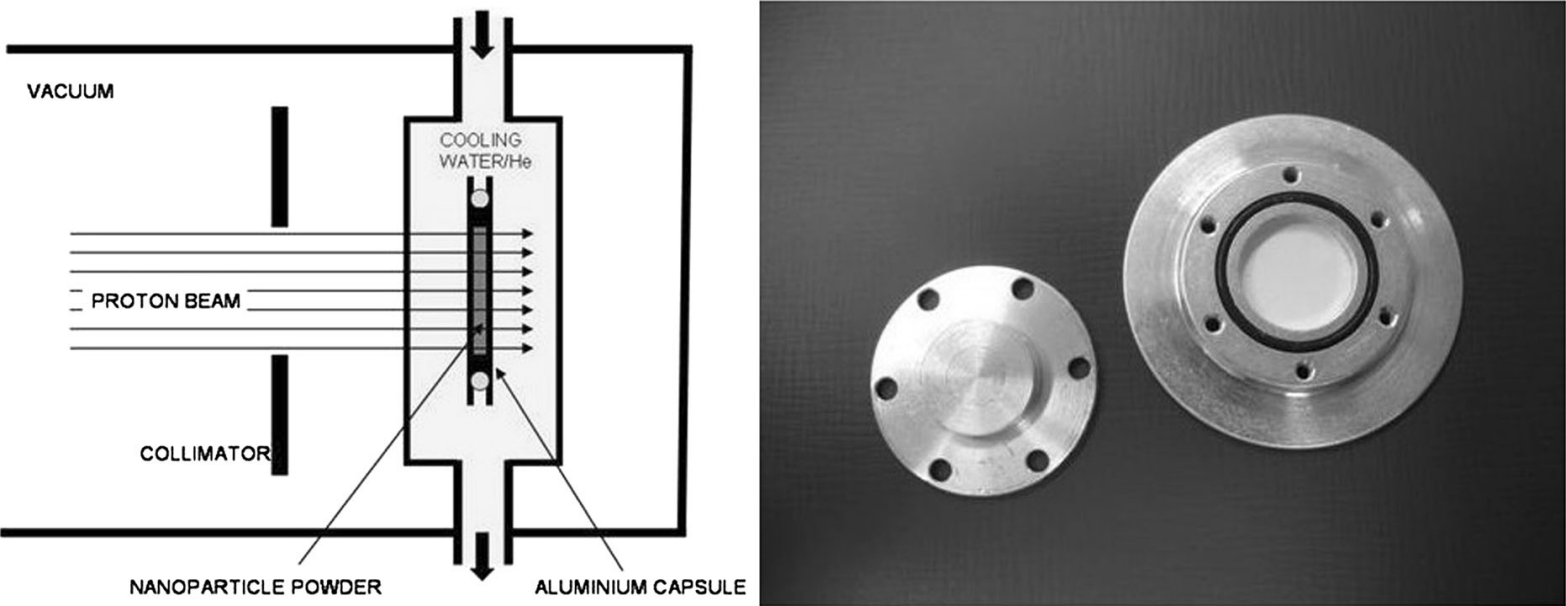
Schematic design of a target system for activating NPs by proton irradiation and photo of an open, powder-filled irradiation capsule. During proton irradiation, the capsule is immersed in cooling water. The proton beam has a diameter of 10 mm defined by a 10 mm collimator in front of the target. The closed irradiation capsule allows the irradiation of a 0.4-mm-thick layer of powder

The target system used for proton irradiations of dry nanoparticle powders has been presented elsewhere (Abbas et al. 2010; Holzwarth et al. 2012). It was designed to overcome the problem of excessive heating in NP samples with poor thermal conductivity by keeping the distance of NPs to cooled surfaces very short. This implies that only thin NP layers can be irradiated. Hence, a flat capsule design with an inner diameter of 10 ~ mm was developed in which a sample of 0.4 mm thickness can be irradiated. A magnesium-alloyed aluminium material (alloy Al 5754) was selected which combines high thermal conductivity and sufficient mechanical strength with low undesired activation of the material. During proton irradiation, the capsules were completely immersed in cooling water. In order to keep the proton energy degradation reasonably low, the beam entrance windows from the beam line vacuum to the target system and from the cooling water into the powder-filled target capsule have a thickness of only 0.3 mm. The cooling water layer is 2.35 mm thick, which is enough to ensure sufficient water flow and cooling while keeping proton energy degradation within acceptable limits. The principle of the system is illustrated in Fig. 2, and a photo of an open, powder-filled irradiation capsule is presented. Typically, batches of 25–30 mg of dry NP material mixed with a lithium compound can be irradiated in such capsules.

### Irradiation conditions

The definition of the irradiation conditions takes into account that the proton energy is degraded by the passage through the aluminium windows, the cooling water layer and in the powder mixture. In order to assure production of ^7^Be in the whole target volume, the proton energy must not fall below the reaction threshold of *E*_thr_ = 1.88 MeV inside the capsule. Hence, an exit energy *E*_p,out _≈ 2 MeV is desirable. Moreover, the energy loss Δ*E*_p_ = *E*_p,in_–*E*_p,out_ of the protons in the target powder must not exceed 3 MeV because the temperature increase in the irradiation capsule is proportional to the product of dissipated beam energy Δ*E*_p_ and the proton beam current *I*_p_. The higher the Δ*E*_p_, the lower is the allowable *I*_p_ leading to excessively long irradiation times to produce useful quantities of ^7^Be. Therefore, an incident proton beam energy of *E*_p,in_ ≈ 5 MeV is desirable. Setting the cyclotron to *E*_p,set_ = 19 MeV allows using a standard setting and gives an incident proton energy of *E*_p,in_ ≈ 4.8 MeV that complies with the requirement. The energy degradation is determined with the widely used simulation programme SRIM (Stopping and Range of Ions in Matter; Ziegler et al. 2013, 1996).

**Fig. 3:**
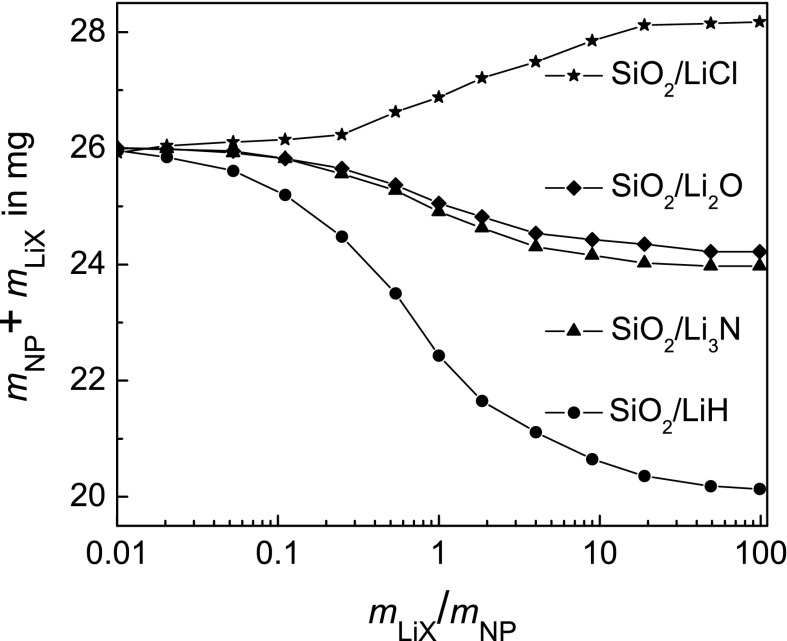
Optimised mass *m*_LiX_ + *m*_NP_ to be filled into the irradiation capsule for different lithium compounds in order to slow down protons from 4.8 to 1.9 MeV depending on the mass ratio *m*_LiX_/*m*_NP_

Further, SRIM simulations are performed to find the proper density of the lithium compound–NP mixture, which is required to reduce *E*_p,in_ ≈ 4.8 MeV to just *E*_p,out_ ≈ *E*_thr_ ≈ 1.9 MeV. For each mixing ratio of lithium compound to NPs, a density value exists that fulfils this condition. This optimised density is adjusted by the mass of the powder mixture loaded into the capsule as presented in Fig. 3. When closing the irradiation capsule with the cover and tightening the screws, the powder will be moderately compressed.

### Nanoparticle recovery and dispersion procedure

#### Nanoparticle recovery

The total activity of ^7^Be produced during irradiation was determined by quantitative γ-ray spectrometry after 2 days of ‘cooling’ in order to allow for the decay of short-lived activation products in the aluminium material of the capsule.

In order to recover the irradiated and radiolabelled NPs, the irradiation capsule was opened in a Petri dish that just houses the external capsule diameter and which was immersed in a minimum volume of water (or absolute ethanol in case of LiH) just covering it in a way to avoid any contact of irradiated powder with air, which might lead to undesired dispersion of NPs.

For the quantities of material (up to about 30 mg) and activities (up to 100 MBq of ^7^Be), it is sufficient to shield the capsule behind 5 cm of lead bricks and to use pincers and pipettes that allow maintaining a minimum distance of about 10–20 cm between radioactive material and hand. Manual skills acquired during training with cold material allow to keep the total exposure to recover and wash the NPs well within acceptable limits.

The complete volume of about 3–5 mL liquid was filled in a vial with a 10 ~ kDa filter and centrifuged for 15 ~ min with a relative centrifugal force of 3,460 ~ *g*. The NPs retained by the filter were twice washed in a mixture of absolute ethanol and demineralised water in a ratio of 50:50 v/v and subsequently 6 times using 0.25 M hydrochloric acid. The washing procedure was finalised with two steps using demineralised water. All ten washing steps were performed with the same filter retaining the NPs, and then they were recovered by back flushing from the filter. For the present study, the water containing the NPs was evaporated under vacuum and mild heating to obtain dry, radiolabelled NPs that can be weighed in order to precisely determine the radiolabelling yield in MBq/mg.

#### Dispersion procedure

After having determined the ^7^Be activity concentration in the dry NPs, the NP powder was dispersed for further characterisation. For this purpose, 2 mg of the dry NPs were dispersed in 20 mL of absolute ethanol (i.e. 0.1 mg/mL). The colloidal solution was mixed and sonicated with an ultrasound probe at 40 % amplitude for 40 min (Digital Sonifier 450 400 W from Branson; corresponding to 2.3 W acoustic power in a total volume of 20 mL) in an ethanol-ice cooling bath in order to avoid excessive heating of the NPs. The dispersion was vortex mixed for 1 min and directly used for DLS and ζ-potential measurements and the preparation of XRD and TEM specimens.

### Quantitative γ-ray spectrometry

Quantitative γ-ray spectrometry was applied to quantify the ^7^Be activity in the irradiation capsule, in the washing liquid during the various washing steps, the ^7^Be-activity retained by the filter after NP recovery and in the finally recovered dried NPs. All activity values were calculated back to end of bombardment as reference time *t* = 0.

Quantitative γ-ray spectrometry was performed with ultrahigh-purity germanium detectors calibrated in energy and efficiency with certified standard calibration sources for each standard geometry. Standardised geometrical conditions were defined for measuring the activity of the irradiation capsules, activity in centrifugation vials with filters, the activity recovered in vials containing standard volumes of suspensions and in vials containing dried recovered NPs or the residual activity left in pipettes. The activity standards had an uncertainty of (1–1.5) % and were supplied from DAMRI and CERCA (France), ENEA (Italy) and the Czech Metrological Institute (Czech Republic).

The efficiency calibration of the γ-ray spectrometers was accurate to within 5 %, and the statistical uncertainty was kept below 1 % by adjusting the counting time. An additional residual uncertainty of about (5–15) % was due to the uncertainty of the activity distribution in the standard geometries. Thus, the ^7^Be activity could be measured with an overall accuracy of about 15 %.

### Nanoparticle characterisation

#### X-ray diffraction

X-ray diffraction analysis was performed using a dedicated glancing-angle X-ray diffractometer (GAXRD), employing Cu K_α_radiation (*λ* = 1.5418 Å) at a tube voltage of 35 keV and current of 30 mA. The diffractometer was equipped with a laser alignment system for the determination of incident angle, an instrumental resolution of about 0.2° and with a solid state detector for resolving the K_α_ radiation and for improving the signal/noise ratio. All GAXRD scans were recorded at an incident angle of 1°. The diffractometer was used in the Grazing Incidence Angle Asymmetric Bragg geometry, which is most suitable for studying polycrystalline surfaces (Gibson 2011). The use of a GAXRD system allows the analysis of very small quantities of NPs.

XRD samples were prepared from suspensions of the recovered SiO_2_ NPs by depositing several droplets of the suspension on a Si wafer. After evaporation of the liquid, the NPs were fixed on the wafer by a drop of PMMA (Poly(methyl methacrylate)) dissolved in anisol. Successfully prepared XRD specimens exhibited a thin and homogeneous spot with a diameter of 5–6 mm on the Si wafer and presented a flat surface for the XRD examinations.

#### Dynamic light scattering and ζ-potential measurements

The hydrodynamic diameter of the NPs was determined by Dynamic Light Scattering (DLS) using a Zetasizer Nano ZS system (Malvern Instruments: Malvern, UK). For this purpose, NPs were dispersed as described previously. After 180 s equilibration at 25 °C, each sample was measured 3 times, and the results were averaged using the instrument’s software. No filtration or centrifugation procedure for size preselection was applied in order to assess the effect of the labelling procedure on the state of aggregation or agglomeration.

The results will be represented as size intensity distribution that presents the rough data of the DLS examination. The conversion to the number distribution weighs the intensity distribution with the size–intensity relationship, which, however, amplifies any uncertainty in the intensity measurement as the scattering intensity is determined by sixth power of the NP diameter *d*_NP_.

In order to determine the possible effect of ^7^Be-recoil labelling on the surface charge of the NPs in suspension, ζ-potential measurements were performed on the as-received and radiolabelled SiO_2_ NPs also using the Malvern Zetasizer Nano ZS system.

#### Electron microscopy

TEM samples were prepared by depositing 4 μL of dispersed solution on carbon-coated TEM grids (carbon type-B, 200 mesh copper grids, supplied by Ted Pella, Inc). Drying in air was accelerated by applying the dispersion protocol described in Sect. Nanoparticle recovery, which used absolute ethanol as solvent.

High-resolution (HR) TEM was performed on a JEOL 2100 microscope operated at an acceleration voltage of 200 kV. Digital images were analysed with the ImageJ software (available at http://rsb.info.nih.gov/ij/). The NP size distribution was determined by image processing of several TEM images in order to evaluate at least 100 isolated primary particles using the interface particle size analyzer (PSA) macro for ImageJ.

#### Leaching tests

In order to assess whether the ^7^Be radiolabels were stably incorporated into the NP structure, leaching tests were performed in demineralised water and with cell culture medium under conditions relevant for in vitro experiments. For this purpose, NP suspensions with a concentration of 6 μg/mL were prepared and 35 mL were filled in 50 mL Falcon tubes, which where then exposed to 37 °C for 6, 24, 48 and 72 h in an incubator. After these time steps, a tube was taken, mixed by inverting it a couple of times, and three aliquots of 2.5 mL were taken and centrifuged with 10 kDa Amicon filters at 6,000 rpm (relative centrifugal force of 3,460×*g*) for 20 min to separate the NPs from the liquid. Then, the activity in the filtrate was determined by γ-ray spectrometry.

## Expected radiolabelling yield

A simple model has been developed to estimate the radiolabelling yield that can be expected from recoil labelling. The basic assumption is that the NPs and the lithium compound are homogeneously mixed. This implies that NPs and lithium compound grains should have the same or a similar size. However, this is difficult to satisfy experimentally. At least the grain size of the lithium compound should be smaller than the range of the recoiling ^7^Be atoms in the lithium compound in order to ensure that the ^7^Be atoms can escape the lithium compound and have the possibility to reach a NP. Careful mixing of the lithium compound with the NPs helps to achieve this since NPs of hard materials such as diamond, alumina or silica will grind the softer lithium salts down to a few μm grain size.

In the energy range *E*_p_ ≤ 10 MeV, the nuclear reaction ^7^Li(p,n)^7^Be proceeds via the formation of a *compound nucleus* (Newson et al. 1957; Krane Krane 1997; Ajzenberg-Selove 1988), which allows a straight forward calculation of the energy of the ^7^Be-recoil atoms as a function of the proton energy *E*_p_ (Marion and Young 1968; Shultis and Faw 2002). The ^7^Be-recoil energy *E*_r_ translates into a recoil range *R*_r_ of up to 6 μm using the data provided by SRIM (Ziegler et al. 2013).

The expected ^7^Be activity after irradiation for a time *t* with protons of an energy *E*_p_ and a proton flux $\dot{\phi }$ per second can be calculated as

1 $$ A(t) = N_{\text{V}} \dot{\phi }\left( {1 - e^{ - \lambda t} } \right)\int\limits_{{E_{\text{thr}} }}^{{E_{{{\text{p}},{ \hbox{max} }}} }} \,\sigma (E_{\text{p}} )\mathop {\left[ {\frac{{{\text{d}}E_{\text{p}} }}{{{\text{d}}x}}} \right]}\nolimits^{ - 1} {\text{d}}E_{\text{p}}, $$

where

2 $$ N_{\text{V}} = \frac{{m_{\text{LiX}} N_{\text{A}} Cf}}{{m_{\text{mol}} V_{\text{c}} }} $$

is the number of lithium atoms per unit volume. Here, *C* denotes the stoichiometry factor for the compound containing the target atom, which is *C* = 1, 1, 2 and 3 for LiCl, LiH, Li_2_O and Li_3_N, respectively. *f* represents the isotopic abundance of the target isotope in the target element, which is *f* = 0.925 for ^7^Li in natural Li. *m*_LiX_ denotes the mass of the lithium compound loaded into the irradiation capsule and *m*_mol_ its molar mass. *V*_c_ is the volume of the irradiation capsule, which is 3.14 × 10^−2^ cm^3^ for the capsule described earlier. The yield is limited in time by the term 1-e^−*λt*^ which tends to 1 for *t* → ∞ when the activity increase is balanced by the decay of the already produced radionuclides, and the activity approaches a saturation value (Podgoršak 2006).

The mixing ratio *m*_LiX_/*m*_NP_ and the mass of the powder mixture mainly affect the number of target atoms *N*_V_, but it also affects the stopping power d*E*_p_/d*x*, which determines the proton energy degradation Δ*E*_p_. When using the optimised densities, i.e. the mass of the powder mixture loaded in the irradiation capsule that just degrades the proton energy from 4.8 to 1.9 MeV (see Fig. 3), the values obtained for the numeric integration in Eq. (1) vary by less than 2 % in the composition range 0.01 ≤ *m*_LiX_/*m*_NP_ ≤ 100.

From the ^7^Be activity, *A*_7Be_ is obtained from Eq. (1), the number of ^7^Be atoms, *N*_7Be_, that have been produced during the irradiation can be calculated as

3 $$ N_{{7{\text{Be}}}} = \frac{{A_{{7{\text{Be}}}} }}{\lambda }, $$

where *λ* is the decay constant of ^7^Be. This corresponds to the number of ^7^Be-recoils that can interact with the NPs during the irradiation.^3^ In analogy to Eq. (1), we can estimate the number of ^7^Be atoms that are implanted in NPs and have become radiolabels as

4 $$ N_{\text{L}} = \frac{{N_{\text{NP}} N_{{7{\text{Be}}}} }}{{V_{\text{c}} }}\int\limits_{0}^{{E_{{{\text{r}},{ \hbox{max} }}} }} \,\sigma_{\text{trap}} (E_{\text{r}} )\mathop {\left[ {\frac{{{\text{d}}E_{\text{r}} }}{{{\text{d}}x}}} \right]}\nolimits^{ - 1} {\text{d}}E_{\text{r}}, $$

where we replace the proton flux by the number *N*_7Be_ of recoiling ^7^Be atoms and the number of target atoms per unit volume by the number *N*_NP_ of NPs present in the target, which can be calculated as

5 $$ N_{\text{NP}} = \frac{{m_{\text{NP}} }}{{\frac{4\pi }{3}\left( {\frac{{d_{\text{NP}} }}{2}} \right)^{3} \rho_{\text{NP}} }}, $$

from the mass *m*_NP_ of NPs loaded into the irradiation capsule and the mass of an individual NP with a diameter *d*_NP_. *ρ*_NP_ denotes the bulk density of the NP material, which gives accurate values for NPs larger than (5–10) nm (Vollath 2008).

**Fig. 4:**
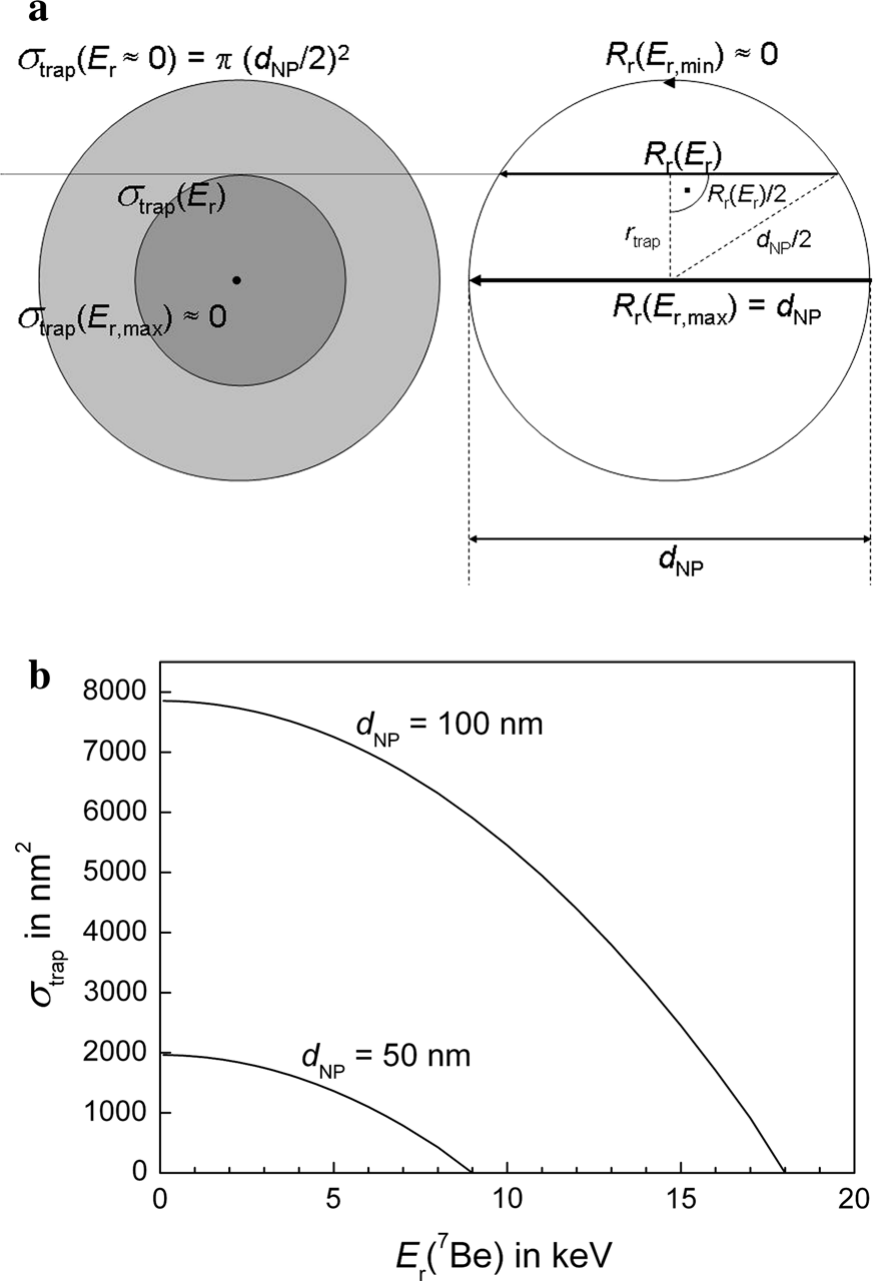
**a**, **b** ^7^Be-recoil atoms with an energy *E*_r_ ≈ 0 can be stopped by the whole NP because of their range *R*_r_ ≈ 0. With increasing recoil energy *E*_r_, their range *R*_r_ increases and the effective trapping cross section decreases from *πd*_NP_^2^/4 to *πr*_trap_^2^. ^7^Be-recoils cannot be stopped by NPs that are smaller than their range (*d*_NP_ < *R*_r_). SRIM data (Ziegler et al. 2013) show that a linear approximation for *R*_r_(*E*_r_) holds for ^7^Be-recoils, which allows to derive in **b** the trapping cross section *σ*_trap_ as a function of the recoil energy *E*_r_ calculated according to Eq. (6)

For the integral in Eq. (4), we have to define a *trapping cross section* *σ*_trap_(*E*_r_) of the NPs for recoiling ^7^Be atoms that takes into account that, due to their small dimensions, NPs can trap ^7^Be atoms only when they are very close to the end of their trajectory, having nearly lost all their recoil energy. As illustrated in Fig. 4a, the highest recoil energy that allows labelling of a spherical NP belongs to the recoil range *R*_r_ that corresponds to the NP diameter *d*_NP_. Therefore, the trapping cross section is increasing from a ‘point’ for *R*_r_ = *d*_NP_ to the full cross-sectional area π*d*_NP_^2^/4 of the nanoparticle when *E*_r_ ≈ 0 and *R*_r_ ≈ 0. The situation for intermediate cases with 0 < *R*_r_ < *d*_NP_ is illustrated in Fig. 4a. A simple dependence of the trapping cross section *σ*_trap_(*E*_r_) on the recoil energy can be introduced by expressing the recoil range *R*_r_ as a linear function of the recoil energy by *R*_r_ = *α* *E*_r_, which is a valid approximation for most materials and recoil ranges in the order of the NP diameter (Ziegler et al. 2013). Therefore, the recoil-trapping cross section of a nanoparticle with diameter *d*_NP_ can be expressed in the range 0 ≤ *r* ≤ *R*_r_ as

$$ \sigma_{\text{trap}} (E_{\text{r}} ) = \pi r_{\text{trap}}^{2} $$

6 $$ = \frac{\pi }{4}\left( {d_{\text{NP}}^{2} - (\alpha E_{\text{r}} )^{2} } \right), $$

where *α* is a constant that can be derived from SRIM data (Ziegler et al. 2013). Consequently, the *E*_r_ dependence of *σ*_trap_ has a parabolic shape dropping from its maximum value *π*(*d*_NP_/2)^2^ for *E*_r_ = 0 to zero at a maximum recoil energy *E*_r_ where *R*_r_ = *d*_NP_ holds as shown in Fig. 4b for two types of SiO_2_ NPs with 50 and 100 nm mean diameter.

One can now calculate from Eq. (4) the expected number *N*_L_ of ^7^Be-recoils trapped in NPs and, hence, the activity concentration *a*(^7^Be) in the NPs as

7 $$ a(^{7} {\text{Be}}) = \frac{{N_{\text{L}} \lambda }}{{m_{\text{NP}} }}, $$

where *λ* denotes the decay constant of ^7^Be. Using Eq. (1) with a proton flux corresponding to a proton current of 1 μA and a duration of 1 hour, we can express *a*(^7^Be) in MBq/μA h mg.

**Fig. 5:**
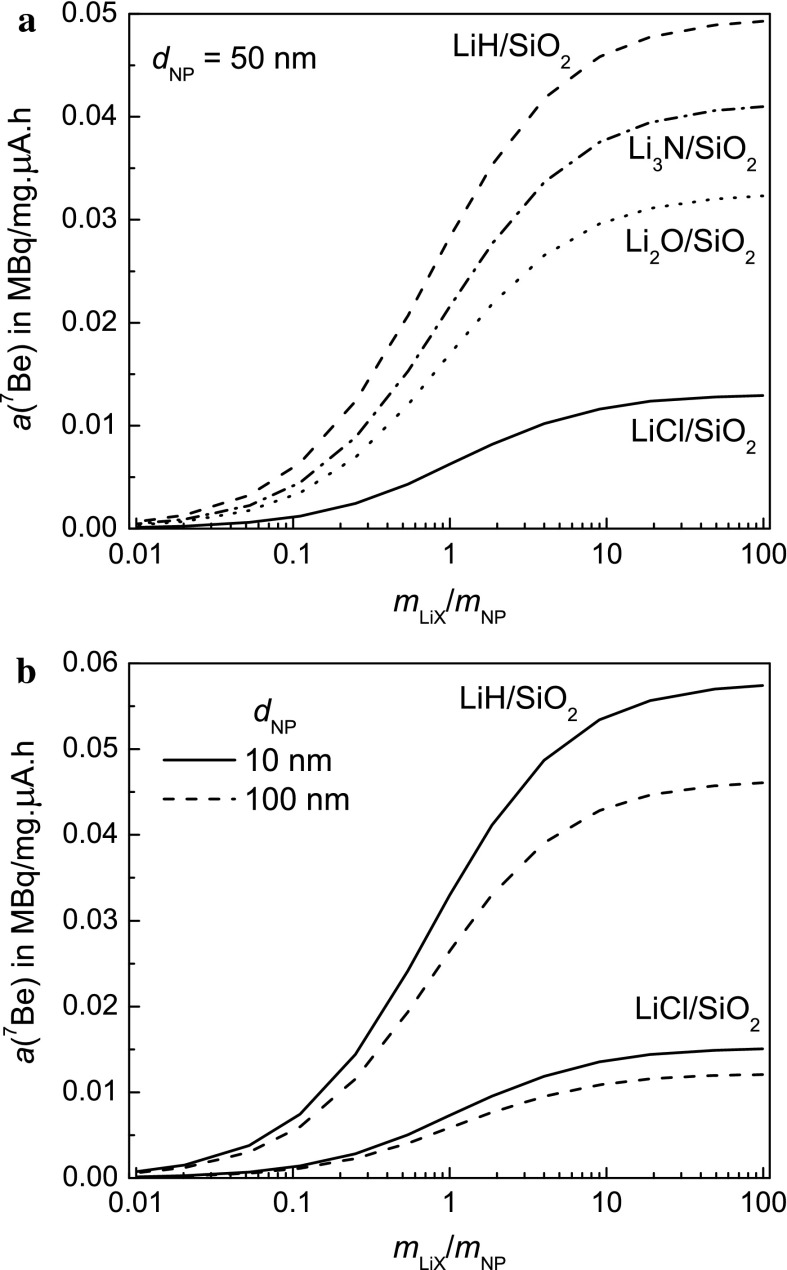
**a**, **b** Calculated ^7^Be activity concentrations *a*(^*7*^Be) given in MBq/μA h mg as a function of the mixing ratio *m*_LiX_/*m*_NP_ for different Li compounds (LiH, Li_3_N, Li_2_O and LiCl) as radiolabel sources. The calculations are made for *d*_NP_ = 50 nm and the optimised values of *m*_LiX_ + *m*_NP_ according to Fig. 3. The differences are mainly caused by the different numbers of Li atoms in the irradiation capsule. In **b**, the effect of NP size on a(^7^Be) is demonstrated for mixtures of SiO_2_ NPs with LiH and LiCl

The model results can only be considered reliable within a factor of two since the stopping power d*E*_r_/d*x* in Eq. (4) refers to the energy degradation the recoiling ^7^Be atoms undergo in the NP Li compound mixture. Dealing with the granularity of this medium (grains size distributions of the lithium compound, size distributions of NPs, homogeneity of mixture) would require extensive simulations. Taking as two extremes, the stopping powers of ^7^Be (i) in amorphous silica (2.2 g/cm^3^) and (ii) in a homogeneous distribution of all atoms in the capsule for the optimised filling density indicate that there is reasonable agreement between both cases for mixtures of SiO_2_ with LiH, Li_2_O and Li_3_N, whereas case (i) defines a lower limit of *a*(^7^Be) in LiCl/SiO_2_ mixtures. All model estimates depicted in Figs. 5 and 7 refer to case (i).

Figure 5a shows the expected activity concentration versus the mass ratio *m*_LiX_/*m*_NP_ of the powder mixture for silica NPs with a diameter of 50 nm with various Li compounds. The activity concentration ‘saturates’ for increasing values of *m*_LiX_/*m*_NP_ > 3 since with increasing mass of the lithium compound a higher flux of ^7^Be-recoils is produced; however, above a certain ratio, the amount of lithium compound surrounding the NPs that can contribute to their labelling cannot be further increased due to the limited range of the recoils in the compound. Figure 5b shows that the activity concentration depends also on the NP diameter.

## Results and discussion

### Activity concentration in ^7^Be-recoil labelled nanoparticles

**Fig. 6:**
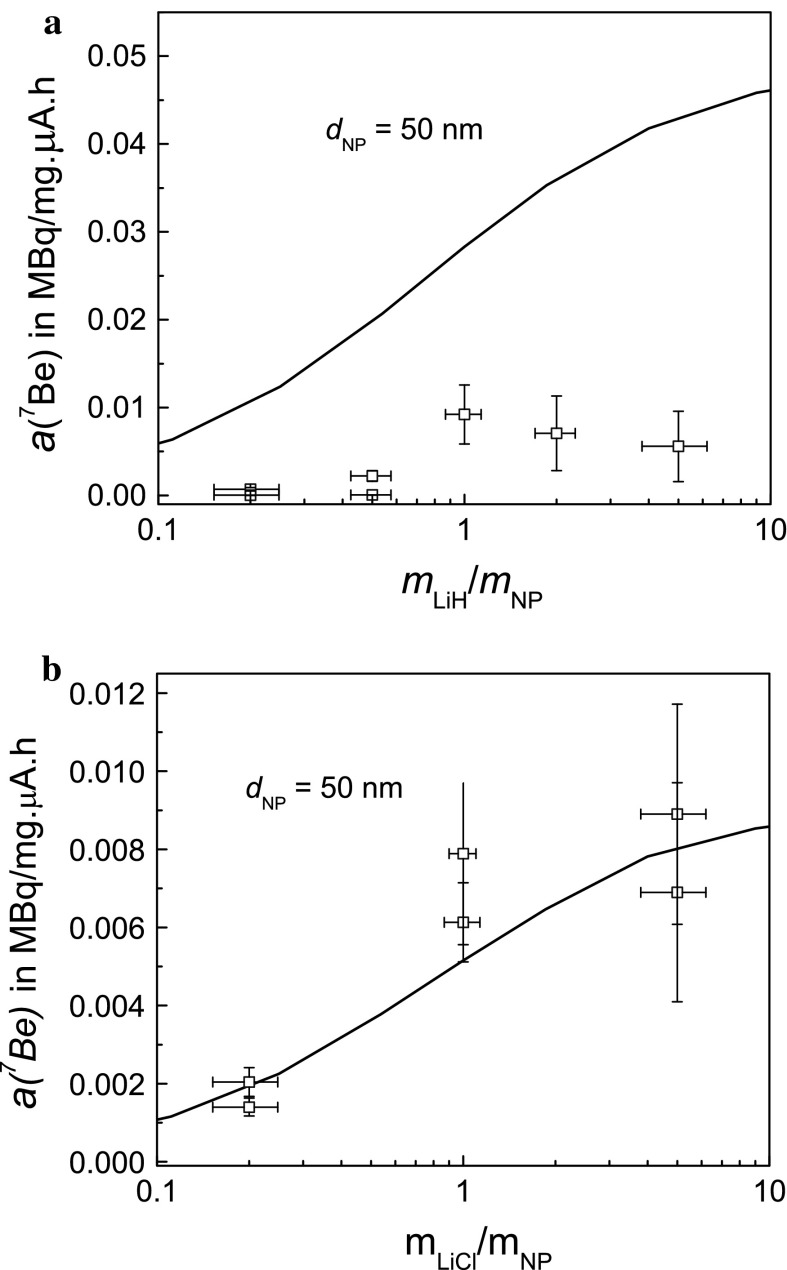
**a**, **b** Experimentally determined activity concentration *a*(^7^Be) given in MBq/mg μA h for industrially manufactured SiO_2_ NPs mixed with **a** LiH and **b** LiCl as a function of the mass ratio *m*_LiH_/*m*_NP_ and *m*_*L*iCl_/*m*_NP_, respectively. The expected values for LiX/SiO_2_ mixtures have been calculated from Eq. (7) for NPs of 50 nm size. Experimental values of LiH/SiO_2_ mixtures are by a factor of 5–10 lower than expected. **b** The results obtained with LiCl/SiO_2_ mixtures match the expectations

**Fig. 7:**
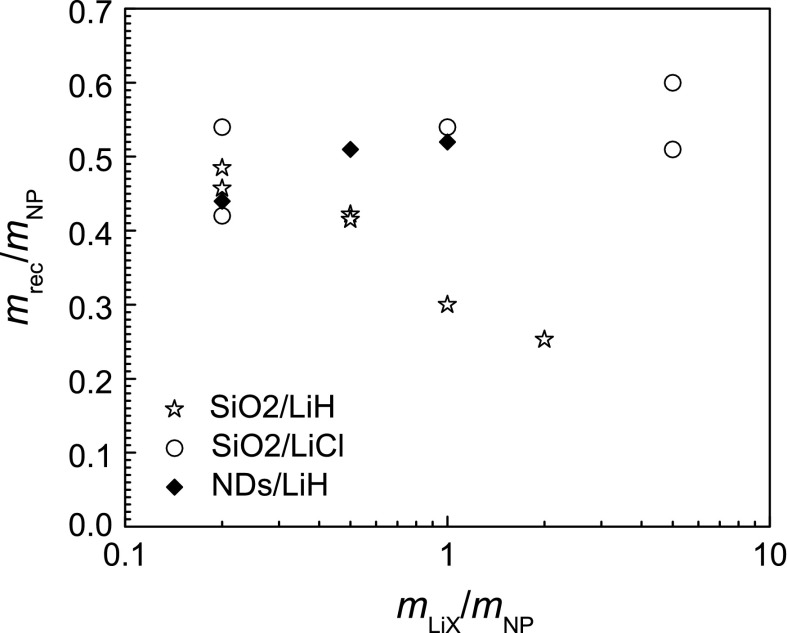
Recovery yields, defined as the ratio of dry recovered NP mass, *m*_rec_, normalised to the mass *m*_NP_ of SiO_2_ NPs loaded into the irradiation capsule. For comparison, results for nanodiamonds (NDs) mixed with LiH are included

The ^7^Be activity concentrations were determined in a series of experiments using SiO_2_ NPs mixed with LiH and LiCl. Figure 6a shows the activity concentrations resulting from an initial series of experiments performed on LiH/SiO_2_ NP mixtures. The obtained values were a factor of 5–10 lower than expected, i.e. by far more than the uncertainty of the model. Additionally, it was found that the fraction of recovered SiO_2_ NPs was unsatisfactorily low. Experiments performed in parallel on nanodiamonds mixed with LiH (not presented here) were, however, in line with expectations. Thus, it was suspected that SiO_2_ NPs could be chemically attacked by immersion in the strongly basic aqueous solution of LiH. The fact that the achieved recovery yields presented in Fig. 7 decreased with increasing LiH content appeared to corroborate this assumption.

Another series of experiments was performed with LiCl/SiO_2_ NP mixtures which resulted in activity concentrations that agree with the expected values as can be seen in Fig. 6b. Usually, about 50 % of the SiO_2_ NPs loaded into the irradiation capsule could be recovered. While this requires further investigation in order to increase recovery yields, the results demonstrate that it is possible to recover 5–10 mg of ^7^Be-labelled, dry SiO_2_ NPs and that an activity concentration of 1 ~ MBq/mg, which is sufficient for most in vitro experiments (Abbas et al. 2010; Gibson et al. 2011), can be obtained with a proton dose of 150–200 μA h. Taking into account that the model expectations presented in Fig. 6b can be considered as a lower limit for LiCl/SiO_2_ mixtures (see Sect. Expected radiolabelling yield), more effort spent on the homogenization of the mixture should even increase the achievable activity concentrations because in lithium-compound grains of several μm size effects of self-absorption of ^7^Be atoms have to be expected.

### Integrity of ^7^Be-recoil labelled nanoparticles

A critical point for all labelling approaches is that the physico-chemical properties of the NPs must be preserved in order to use them as tracers in meaningful experiments to characterise cell uptake or biodistribution. In the present case, recoil radiolabelled particles were characterised by XRD, DLS, ζ-potential measurements and TEM.

**Fig. 8:**
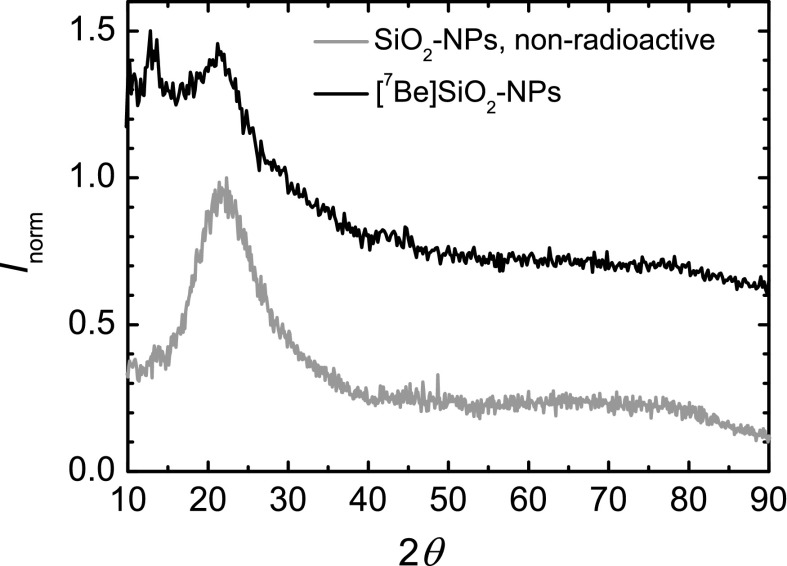
X-ray diffraction scans (normalised intensity versus 2*θ* for the as-received and ^7^Be-recoil labelled SiO_2_ NPs (*m*_LiX_/*m*_NP_ = 1, proton dose 125 μA h.). The poorer signal/noise ratio for the radiolabelled NPs is due to the lower amount of material used for XRD. The peak around 17.5° is due to the PMMA used to fix the NPs on the Si wafer

For amorphous SiO_2_, XRD has only a limited use since an XRD scan shows only one broad maximum. However, if any significant fraction of the material would crystallise under the combined effect of proton irradiation and the created flux of recoiling and sputtered atoms in the powder mixture, the scan would indicate early the presence of some crystal diffraction peaks, even if it would still be difficult to quantify the effect. The XRD scans before and after ^7^Be-recoil labelling, presented in Fig. 8, give no indication of increased crystallinity. These scans were in fact made in the as-received and recoil-labelled NM203 silica nanomaterial, with approximately same primary particle size as NM200 (Rasmussen et al. 2013), under more extreme irradiation conditions.

**Fig. 9:**
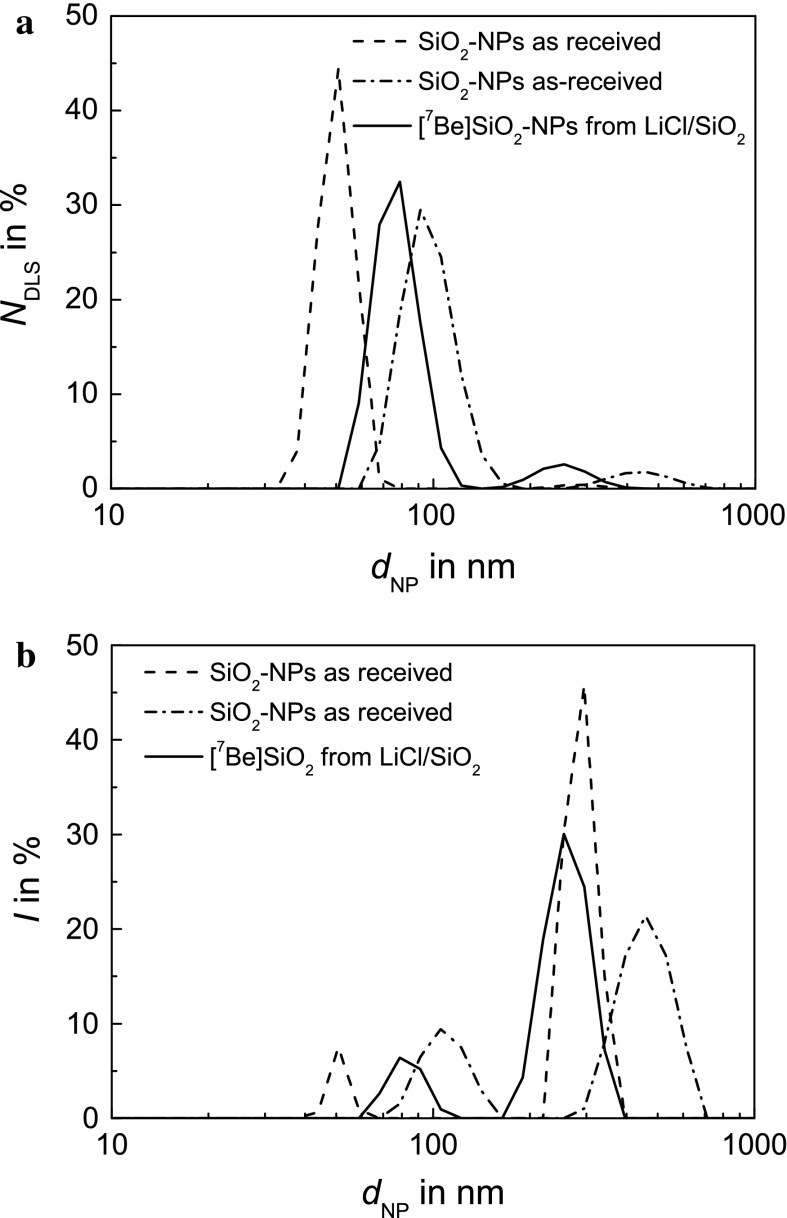
**a**, **b** Hydrodynamic size of SiO_2_ NPs before and after ^7^Be-recoil labelling in a SiO_2_ NP/LiCl mixture (*m*_LiCl_/*m*_NP_ = 1). DLS results are presented as **a** number–size distribution and **b** size–intensity distribution. The results for ^7^Be-labelled NPs are in the range of the as-received NPs

The effect of ^7^Be-recoil labelling on the hydrodynamic size of the SiO_2_ NPs was determined by DLS. The results presented in Fig. 9 show that ^7^Be-labelled SiO_2_ NPs can be dispersed after recovery, washing and drying, giving results in the range of the industrially manufactured base material, provided radiolabelling was done in a mixture with LiCl. After radiolabelling by proton irradiation of a LiH/SiO_2_ mixture, large aggregates of more than 1,000 nm in size dominate the DLS, and the results are not comparable with the non-labelled material. The numeric evaluations of the DLS experiments are compiled in Table 2. In Fig. 9 and Table 2, two sets of measurements are presented for the as-received, non-labelled material. They illustrate the variability of the DLS results even if the same dispersion protocol is applied on 2 mg of NM200 SiO_2_ NPs sampled from the same vial containing 2 g of material. Thus, the result presented for the SiO_2_ NPs after having been irradiated in a LiCl/SiO_2_ mixture is in the range to be expected for the material, and we may, therefore, conclude that the ^7^Be-recoil labelling procedure does not alter the properties of the suspensions that can be obtained. The same can be stated for the ζ-potential (see Table 2). However, since DLS indicates the existence of very large aggregates after ^7^Be labelling in LiH/SiO_2_ NPs mixtures, the ζ-potential data obtained for NPs radiolabelled by the use of LiH are not comparable to the other data.

**Table 2:** Comparison of the DLS results and the ζ-potential measurements of the as-received SiO_2_ NPs and ^7^Be-recoil labelled SiO_2_ NPs radiolabelled by proton irradiation of nanoparticles mixed with the same mass of LiH and LiCl

Material	^7^Be source	DLS results	*I*_DLS_		*N*_DLS_		PDI	ζ-pot
			nm	%	nm	%		mV
SiO_2_ (type NM200) as received	n.a.	Peak 1	51	9	50	99	0.528	−31
		Peak 2	290	91	287	1		
SiO_2_ (type NM200) as received	n.a.	Peak1	109	28	98	93	0.649	−29
		Peak2	462	72	445	7		
[^7^Be]SiO_2_ recoil labelled	LiH	Peak 1	1,244	100	1,187	100	0.393	−30
		Peak 2						
[^7^Be]SiO_2_ recoil labelled	LiCl	Peak 1	83	15	77	92	0.656	−20
		Peak 2	263	85	255	8		

Two peaks were usually identified in the size intensity (*I*_DLS_) and number size (*N*_DLS_) distributions which are given by their location (in nm) and their intensity (in  %). Presented is also the polydispersity index (PDI)

**Fig. 10:**
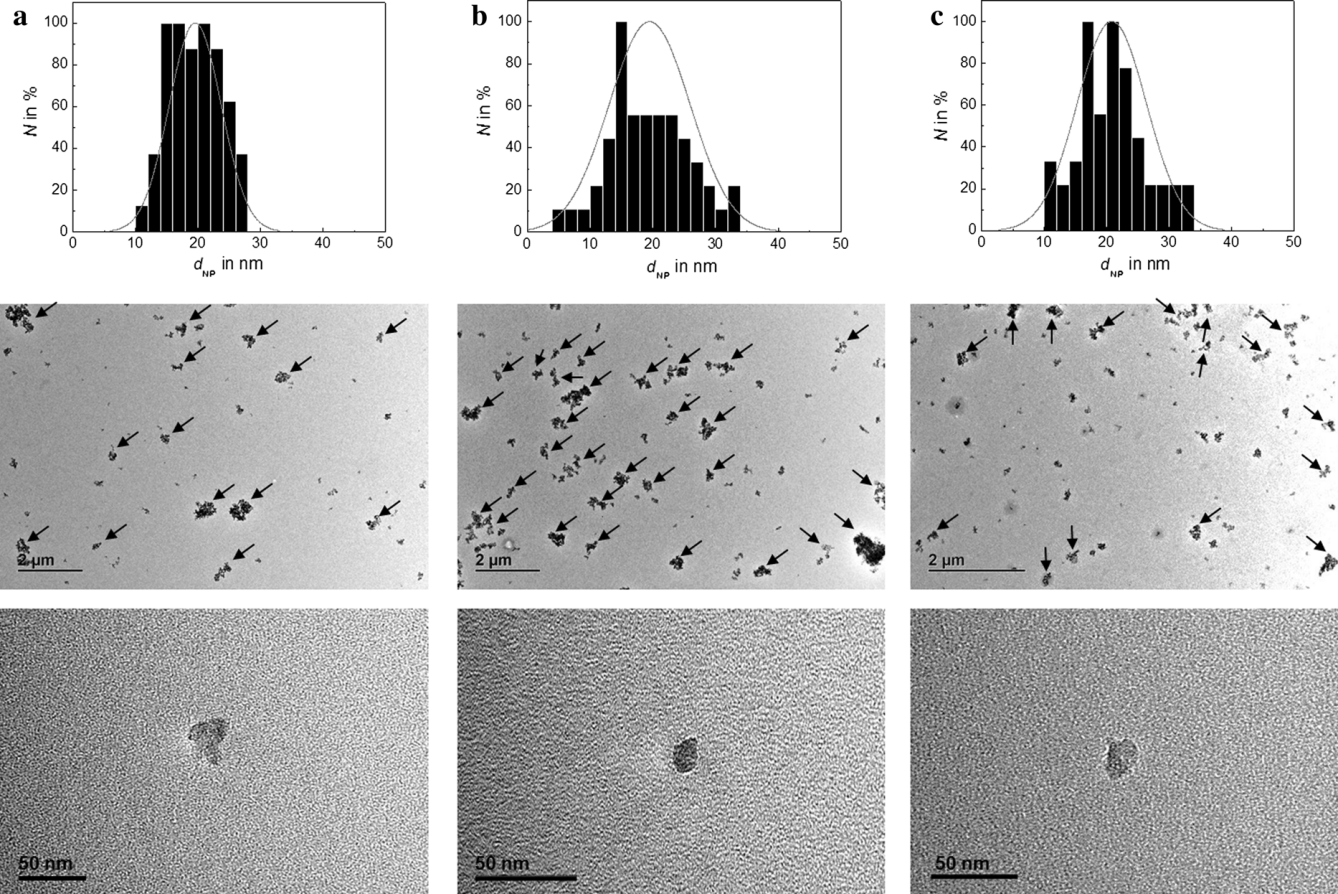
**a**–**c** TEM examination of **a** the as-received SiO_2_ NPs and ^7^Be-recoil labelled SiO_2_ NPs after proton irradiation of a mixture of SiO_2_ NPs with **b** LiH and **c** LiCl. The size distribution has been determined by analysis of at least 100 primary NPs. The overview TEM micrographs show a strong tendency to form larger aggregates after irradiation of a SiO_2_ NP/LiH mixture as compared to the as-received SiO_2_ NPs (**a**) and irradiated SiO_2_ NP/LiCl mixtures (**c**). In order to illustrate the effect of proton irradiation of on SiO_2_ NP/LiH mixtures, larger aggregates/agglomerates, larger than about 200 nm, have been labelled with *arrows*. They are more frequent in (**b**)

In Fig. 10, TEM reveals that the NPs radiolabelled in LiCl/SiO_2_ mixtures are very similar to those of the industrially manufactured base material, whereas radiolabelling in LiH/SiO_2_ mixtures indicates much larger agglomerates or aggregates as evidenced by DLS. However, also in the last case, primary particles can still be found that could quantitatively be analysed by imaging software. The histograms for isolated primary particles give no indications for severe changes.

An important prerequisite for using radiolabelled NPs as tracers is the stability of the radiolabel in the NP, which has been tested under conditions typical of in vitro experiments for up to 72 h in cell culture medium as well as in demineralised water according to the procedure given in Sect. Leaching test using NPs radiolabelled in LiCl/SiO_2_ NP mixtures. In all experiments, the release was below the detection limit for γ-ray spectrometry. This means that under the present experimental conditions and for our spectrometry equipment that with 95 % confidence, the ^7^Be release during 72 h was below 30 ppm. Therefore, the recoil-labelled NPs appear to be stably labelled under all relevant experimental conditions.

### Further improvements and limitations of the method

An increase of the recovery yield presented in Fig. 7 is one of the most desirable improvements. The most likely problem is related with the complete recovery of the NPs when collecting the liquid used for the capsule opening in a Petri dish with a pipette. Incomplete back flushing of NPs retained on the filter or adhesion to the vials appears to be less important as can be inferred from the moderate quantities of radioactivity detectable on the backflushed filters. However, all processing steps need to be improved in order to avoid accumulation of small losses in the whole series of processing steps.

The results clearly show the superiority of using LiCl as source of ^7^Be labels. The mixture of LiCl with SiO_2_ NPs can be irradiated without damage to the NPs, and dry NPs can be recovered. Since the success of the method depends on the homogeneity of the mixture and since the currently applied manual mixing is cumbersome, a new procedure will be tested by mixing the NPs with saturated aqueous LiCl solution and evaporating the water before proton irradiation. Additionally, this method should yield improved thermal contact of the NPs with their surrounding, and it may be expected that the NPs are better protected against the effects of high temperatures during proton irradiation. Analogously, FePt NPs have been protected in a salt matrix to perform annealing treatments at high temperatures to tune their magnetic properties (Farahmandjou 2009; Li et al. 2006). This method could enable using higher proton beam intensities without the risk of thermally induced aggregation of NPs. Consequently, the duration of the proton irradiations could be reduced, and higher activity concentrations could be achievable. It is also likely that the irradiation of thicker NP layers will become possible which enables the production of larger batch sizes of ^7^Be-recoil labelled NPs.

While the macroscopic temperature profile inside the irradiation capsule that is caused by the dissipated proton energy Δ*E*_p_ = *E*_p,in_–*E*_p,out_ can be adjusted by the beam intensity *I*_p_ (Holzwarth et al. 2012), individual NPs are subjected to additional localised microscopic heating for periods of the order of 100 ns (calculated after Lehtinen and Zachariah 2001) when they are passed by a ^7^Be-recoil or when they stop a ^7^Be-recoil and become radiolabelled. The maximum energy transfer of a recoiling ^7^Be atom to a NP depends on the recoil energy *E*_r_, the stopping power d*E*_r_/d*x* in the NP material and the NP diameter *d*_NP_. The dissipated recoil energy in a NP is given by Δ*E*_diss_ = (d*E*_r_/d*x)*· *d*_NP_, and the related temperature increase Δ*T* is higher for smaller NPs as the energy is distributed over a smaller number of atoms. It can be calculated as

8 $$ \Delta T = \frac{{\frac{{{\text{d}}E_{r} }}{{{\text{d}}x}}d_{\text{NP}} }}{{c_{\text{p}} \rho_{\text{NP}} d_{{{\text{N}}P}}^{3} \frac{\pi }{6}}}, $$

using the specific heat capacity *c*_p_ and the density *ρ*_NP_ of the NP (bulk)material. In small NPs, the resulting temperature may exceed the melting point of the material. The effect should generally be tolerable for NPs with diameters above 5–10 nm. In the sense of Eq. (8), *d*_NP_ is the size of the aggregates of smaller primary NPs that allow a rapid distribution of the dissipated energy among all atoms. Assuming that the NPs exchange thermal energy with their environment mainly by heat conductivity of the air trapped in the voids in the powder, it can be estimated that individually heated NPs will thermalise within less than 100 ns with their environment (Lehtinen and Zachariah 2001). During an irradiation to achieve an activity concentration of 1 MBq/mg, a nanoparticle may be subjected about 25 times to such a temperature increase caused by the passage of a ^7^Be-recoil, and it will always thermalise within less than 100 ~ ns. From positron annihilation experiments during isothermal annealing of amorphous bulk silica, it is known that a temperature of 1,500° C has to be hold for at least 300 s before changes of the free volume in the amorphous matrix become detectable (Hugenschmidt et al. 1997). In spite of the small dimensions of SiO_2_ NPs, it is unlikely that thermal effects caused by recoils may alter the properties of the SiO_2_ NPs during ^7^Be-recoil labelling. While even severe damage to individual NPs (e.g. some of the smallest in the size distribution) cannot be completely excluded, the presented experimental results do not support such concern.

Residual lithium impurities could be considered as a drawback for biological or toxicological applications of ^7^Be-recoil labelled NPs. They can be created as a product of atomic displacements following collisions of Li atoms with protons or ^7^Be-recoils. The contribution of displaced Li atoms can be estimated by SRIM simulations (Ziegler et al. 2013, 1996), which yield the number of Li vacancies, i.e. the number of displaced Li atoms in the powder mixture. Such simulations show that ^7^Be-recoils may create about 60 displaced Li atoms. Compared with this number, the effect of proton-induced collisions and collision cascades can be neglected. Based on this number, a Li contamination in the range (100–200) μg/kg can be estimated. Li impurities caused by diffusion effects in the powder mixture can be simulated by furnace experiments (200 °C for 12 ~ hours) and subsequent analysis by Inductively Coupled Plasma Mass Spectrometry (ICP-MS). Such experiments yielded Li contamination levels in the range of (20–60) μg/kg. Hence, a total level of Li impurities of the order of (200–250) μg/kg might be expected, which appears acceptable in in vitro and in vivo experiments due to the low toxicity of Li (Aral and Vecchio-Sadus 2008).

## Conclusions

Industrially manufactured NPs can be radiolabelled with ^7^Be by exposure of dry NP powder mixed with fine-grained LiCl to a proton beam. Labelling occurs in a fraction of the NPs by implantation of recoiling ^7^Be atoms produced via the nuclear reaction ^7^Li(p,n)^7^Be. Batches of about 10 mg of NPs can be radiolabelled, and activity concentrations of 1 MBq/mg can be reached within acceptable irradiation times. Characterisation of NPs post irradiation by DLS, ζ-potential measurements, XRD and TEM gives no indication of irradiation-induced alterations of the NPs’ properties.

With a slight modification of the target system used for the present experiments, ^7^Be-recoil labelling could be performed with cyclotrons with a maximum proton energy of (17–18) MeV which are available in most medical research centres. Due to the rather long half-life of ^7^Be (53.29 ~ days), the proton irradiation can be fractionated over several periods in which the cyclotron is not used for other purposes until the desired activity concentration is reached.

Further improvements of the method may be possible by mixing NPs with a saturated aqueous solution of LiCl and subsequent evaporation of the water. It is expected that embedding NPs in such a salt matrix leads to more homogeneous specimens for proton irradiation and improved thermal contact of the NPs. This should facilitate cooling during irradiation and enable the use of higher proton beam currents. Thus, shorter irradiation times and/or higher activity concentrations should become achievable.

## Footnotes

1 Assuming that Au NPs shall be radiolabelled with ^195^Au produced by the reaction ^197^Au(p,3n)^195m^Hg and the subsequent decay of ^195m^Hg to ^195^Au, the maximum range of recoiling mercury atoms in gold is about 50 nm. Thus, most labels will stay in the NPs, and they have been produced for NPs of at least 50 nm size. Only those recoils produced close to the surface with a direction towards the surface can escape the NP. The smaller the NPs, the more the ^195m^Hg-recoils will be ejected from the NP in which they have been created.

2 In nuclear physics, the reaction cross section describes the size of the projected area around a target nucleus 'T' in which the passage of a projectile 'p' will cause a nuclear reaction leading to a product nucleus 'P' accompanied by the emission of one or more secondary particles 's'. This leads to the notion for nuclear reactions of the type 'T(p,s)P'. The larger the related reaction cross section, the more probable is the nuclear reaction. The unit of the reaction cross section is the barn (1 barn = 10^−24^cm^2^), with cross sections frequently expressed as millibarn (mb).

3 Since the irradiation time is much shorter than the half-life of ^7^Be, decay during the irradiation has been neglected..
